# Ageing across the great divide: tissue transformation, organismal growth and temperature shape telomere dynamics through the metamorphic transition

**DOI:** 10.1098/rspb.2022.2448

**Published:** 2023-02-08

**Authors:** Pablo Burraco, Miguel Hernandez-Gonzalez, Neil B. Metcalfe, Pat Monaghan

**Affiliations:** ^1^ School of Biodiversity, One Health and Veterinary Medicine, University of Glasgow, Glasgow G12 8QQ, UK; ^2^ Ecology, Evolution and Development Group, Doñana Biological Station (CSIC), 41092, Seville, Spain

**Keywords:** climate warming, ectotherms, frogs, growth rate, senescence, *Xenopus*

## Abstract

Telomere attrition is considered a useful indicator of cellular and whole-organism ageing rate. While approximately 80% of animal species undergo metamorphosis that includes extensive tissue transformations (involving cell division, apoptosis, de-differentiation and *de novo* formation of stem cells), the effect on telomere dynamics is unknown. We measured telomeres in *Xenopus laevis* developing from larvae to adults under contrasting environmental temperatures*.* Telomere dynamics were linked to the degree of tissue transformation during development. Average telomere length in gut tissue increased dramatically during metamorphosis, when the gut shortens by 75% and epithelial cells de-differentiate into stem cells. In the liver (retained from larva) and hindlimb muscle (newly formed before metamorphosis), telomeres gradually shortened until adulthood, likely due to extensive cell division. Tail muscle telomere lengths were constant until tail resorption, and those in heart (retained from larva) showed no change over time. Telomere lengths negatively correlated with larval growth, but for a given growth rate, telomeres were shorter in cooler conditions, suggesting that growing in the cold is more costly. Telomere lengths were not related to post-metamorphic growth rate. Further research is now needed to understand whether telomere dynamics are a good indicator of ageing rate in species undergoing metamorphosis.

## 1. Introduction

Understanding the mechanistic causes of ageing is one of the major biological and medical challenges facing us today. Although the shortening of telomeres (the end caps of the chromosomes) is often presented as a marker of ageing and has been shown to be related to individual health and life expectancy in a range of taxa [1], this process still deserves further exploration. Increasing evidence also indicates that the rate of telomere shortening is influenced by the harshness of the environment an individual experiences [2,3], which seems to be driven by an induced oxidative stress state and/or by increases in the rate of cell division [2,4]. However, despite 80% of all animal species undergoing metamorphosis, which involves dramatic changes in body form, there have been no previous studies of the role of this spectacular developmental transition in shaping telomere dynamics. This is especially surprising given the complex patterns of tissue loss, formation and remodelling that occur during this process. Such knowledge would improve our understanding of whether different organs of the same animal show parallel changes in telomere lengths across metamorphosis, thus retaining some legacy of previous cell division pattern, or whether this is dependent on the relative degree of tissue transformation.

Metamorphosis is an ancient life-history trait across the tree of life that involves the transition from a larva to a juvenile life stage [5–7]. This process can reduce intraspecific competition for resources and is known to be a key innovation driving diversification in some taxa (e.g. [8,9]). From a developmental perspective, metamorphosis is considered incomplete when a larva (often called nymph) transforms into an adult phenotype through slow and subtle morphological changes, or complete when abrupt and rapid larval transformations lead to a markedly different juvenile or adult. In vertebrates, the life cycle of many amphibians, particularly anurans, presents a notable example of complete metamorphosis. Anuran metamorphosis generally lasts a few days and involves extensive changes required for the transition from a tail-swimming larva to a tetrapod juvenile, normally also involving habitat and dietary shifts [5]. The process includes the formation of new structures such as limbs, the loss of others such as the tail, and the re-modelling of tissues such as the gut. Other tissues such as liver and heart mostly develop during the larval stages and are carried through to the post-metamorphic stage, although they experience some functional changes across metamorphosis [5,10,11]. At the cellular level, metamorphosis requires extensive cell division and cell death, as well as *de novo* formation of stem cells [5,12]. Although all these processes are thought to affect telomere length [13,14], there have been no previous studies of telomere dynamics across tissues and developmental stages of metamorphosing animals.

Organisms growing at higher rates, including ectotherms, normally experience faster telomere shortening [15,16], a process linked to the oxidative damage caused by the overproduction of reactive oxygen species (ROS), and to higher cell division rates [4,17]*.* Ectotherms often exhibit a remarkable ability to alter their body size in response to environmental conditions such as temperature or desiccation [18,19]. In species with complex life cycles, size at metamorphosis is considered a good predictor of fitness, as it often positively correlates with subsequent survival probability, fecundity or size at maturity [20–22]. In these species, the relationship between growth rate and telomere maintenance is, therefore, expected to be negative throughout the growth period, and be shaped not only by costs of altering growth trajectories in response to environmental changes, but also by the increased energy demands and cellular processes that take place during metamorphosis.

Here, we investigated telomere dynamics across metamorphosis in African clawed frogs (*Xenopus laevis*) exposed to either cool or warm temperatures (19°C or 23°C) during the larval period. *Xenopus laevis* is a fully aquatic amphibian species with a complex life cycle, native to southern Africa, and commonly used as a model organism in molecular and developmental biology. Higher temperatures are known to increase the growth and developmental rates of amphibians, including *X. laevis* [23], and, therefore, are also expected to accelerate the rate of telomere attrition [16,24,25]. Throughout development from larva to adult frog, we measured how telomere lengths changed in five contrasting tissues (tail muscle, gut, heart, liver and hindlimb muscle). These tissues were chosen because they differ in the extent of their transformation from the larva to the post-metamorphic form. For example, from early larval stages, tail muscle is formed and only experiences subtle increases in size until resorption during metamorphosis [26]. By contrast, the gut persists through metamorphosis but is transformed, shortening by 75% and undergoing extensive remodelling that includes the apoptosis and de-differentiation of epithelial cells to stem cells [27–29]. The heart and liver remain relatively unchanged in structure from middle larval stages, but greatly increase in size from metamorphosis onwards [11,30]. Finally, the development of hindlimb muscle includes the fusion of primary larval myoblasts to generate multinucleated myofibres [31,32].

Given the contrasting degrees of transformation across the five studied tissues, we expected different telomere dynamics among them. We hypothesized that telomere lengths in the gut would experience the greatest degree of change through metamorphosis, and particularly that the extensive presence of stem cells could potentially result in longer telomeres. Stem cells are recruited to facilitate the tissue remodelling and renewal, and can have more telomerase activity (the main enzyme responsible for restoring telomeres) and a slower rate of telomere loss, hence their expected longer telomeres [33,34]. We also predicted that cell division across life stages would be associated with gradual shortening of telomeres in liver, heart and hindlimb, while tail muscle telomeres would show the least change over time since tail only experiences small changes in size until resorption. We further predicted a negative relationship between an individual's growth rate and the resulting telomere lengths in its tissues during the larval period, as a consequence of the metabolic demands of both tissue transformation and metamorphosis. We expected that temperature would affect the cost of growth, since higher temperatures potentially allow the animal to digest food faster but also might lead to impaired mitochondrial efficiency and/or greater rates of oxidative damage and so more need for tissue repair [35]. Finally, we predicted that the strength of correlations in telomere lengths across an individual's tissues would be shaped by differences in the degree of tissue transformation across developmental stages, which can include differences in tissue growth and stem cell recruitment.

## 2. Material and methods

### (a) Experimental design and sampling procedure

We obtained five wild-type clutches of *X. laevis* from the European *Xenopus* Resource Centre (University of Portsmouth, United Kingdom; https://xenopusresource.org/about_lines). Embryos were maintained in 10 l tanks filled with 2 l of dechlorinated water at a density of approximately 300 individuals per tank and 19°C (i.e. the temperature used by the *Xenopus* provider) with a light : dark cycle of 12 : 12 h until hatching. Once hatched, we placed 18 larvae from a single clutch in one of eight 10 l tanks (*N* = 8 replicate tanks per clutch; total of 40 tanks; 720 individuals in total). The remaining hatched individuals were euthanized using a buffered MS-222 solution (2 g l^−1^). Half of the tanks (*N* = 20; 4 per clutch) were maintained at 19 ± 0.3°C (hereafter ‘cool treatment’) by a water recirculating system that controlled water temperature as well as providing filtration. The other half were linked to a separate recirculating water system in the same aquarium, in which the temperature was gradually increased from 19°C to 23°C at a rate of 0.5°C/hour to allow acclimation. The temperature was then maintained at 23 ± 0.3°C (hereafter ‘warm treatment’) until half of the larvae reached the onset of metamorphosis (i.e. stage NF60 [36]). At that life stage, the temperature in these tanks was gradually decreased from 23°C to 19°C at a rate of 0.5°C/hour. These two temperatures are within the natural temperature range of *X. laevis* [37]. Therefore, from the onset of the metamorphosis (NF60) onwards, all individuals in the experiment were kept in the same thermal conditions (i.e. 19°C; electronic supplementary material, figure S1). Larvae were fed twice daily with a 1 : 1 mix of Spirulina and Sera Micron powders (Sera Heinsberg, Germany) diluted in water. Once metamorphosed, froglets were fed daily with protein-sinking trout pellets. The amount of food was adjusted throughout the experiment to allow all animals to feed *ad libitum*.

We collected the required tissue samples from individuals at five different stages: (i) approximately at the middle of the larval development (stage NF54, hindlimbs in paddle stage), (ii) at the onset of metamorphosis (stage NF60), (iii) at the end of metamorphosis (stage NF66), and in (iv) 70-day-old and (v) 7-month-old post-metamorphic individuals (see schedule of tissue sampling in electronic supplementary material, figure S1). In this species, individuals can reach sexual maturity when they are 6–8 months old [38], and so we refer to the 7-month-old individuals as adults. We collected three individuals from each tank at NF54, and two individuals from each tank in the successive sampling points. When possible we only used tissue samples from a single individual per tank (the remaining tissues were used for other purposes), except at stage NF54 where we pooled samples from two individuals in order to get enough heart and liver tissue for telomere measurements (see electronic supplementary material, table S1 for sample sizes). After the 70-day sampling, frog density was adjusted to three individuals in each tank to accomplish bioethical guidelines. Development was highly synchronized between individuals within each temperature treatment as overall approximately 85% of individuals reach the targeted developmental stages within a very short period of time (see below). As expected, temperature altered the timing of larval development, thus there was a difference in age at sampling between the temperature treatments: larvae from the 19°C and 23°C treatment took respectively, on average, 25 and 21 days (range ± 1 day) from hatching to reach stage NF54, 42 and 33 days (±2 days) from hatching to reach NF60, and 57 and 47 days (±2 days) from hatching to reach NF66. Therefore, the duration of metamorphosis was similar regardless the temperature experienced during the larval period—as expected since all individuals underwent metamorphosis at the same temperature (19°C). At the post-metamorphic stages, we sampled individuals at the same chronological age, i.e. 70-day- and 7-month-old frogs. In all sampled individuals, we recorded body mass to the nearest 0.01 g. Tissue samples for telomere measurements were first placed in dry ice and then stored at −80°C until assayed (for less than six months). All of the experimental procedures here conducted—including euthanasia—complied with the UK legislation for animal experimental work and the study was approved by the University's Animal Welfare & Ethics Review Board.

### (b) DNA extraction

DNA was extracted using the Puregene DNA extraction kit (Qiagen), following the manufacturer's protocol. This commercial kit enables purification of high-molecular weight DNA (100–200 kb). DNA concentration and quality were quantified using a Nanodrop-8000 spectrophotometer. We checked DNA integrity in a subset of approximately 11% of samples, using an Agilent 2200 TapeStation. Genomic DNA samples were stored at −20°C until assayed (for less than three months).

### (c) Sex determination

In *X. laevis*, sex determination follows a ZW gametic system with heterogametic (ZW) females and homogametic (ZZ) males. The DM domain gene linked-W chromosome (DM-W) is considered to be the master female sex determination gene in this species [39]. To amplify this gene and so determine the sex of each sampled individual, we used the protocol developed by [39] following the recommendations of [40]. In all cases, we used DNA extracted from liver, except in individuals sampled at NF54 for which we used tail muscle (since liver samples had to be pooled from two individuals). See electronic supplementary material for further details.

### (d) Telomere analysis

As in other vertebrates, *X. laevis* telomeres comprise tandem repeats of the TTAGGG sequence at the terminal regions of chromosomes [41]. The *X. laevis* genome is characterized by the almost total absence of interstitial repeats of the telomere sequence [41,42], which makes this species a good candidate to measure telomeres using qPCR. For telomere length quantification, we followed a standard qPCR procedure used in many other vertebrate species and taxa [43] to estimate relative telomere length using the threshold cycle value (Ct) of telomeric repeats and a reference gene designed for *X. laevis* with non-variable copy number (RAG). See electronic supplementary material for further details.

### (e) Statistical analyses

All statistical analyses were conducted in R (R Development Core Team 2019, version 3.6.1). To check for a possible effect of temperature on survival across developmental stages, we ran a generalized mixed model with a Poisson distribution (*glmer* function in package *lme4*), including the *number of individuals alive at each sampling point* as the dependent variable, *temperature* and *developmental stage* (and their interaction) as independent variables, and *tank* nested within *clutch* as random factors. Since the temperature manipulation induced differences in body mass in addition to changes in developmental timing across larval stages, we calculated individual growth rate as a parameter integrating the amount of body mass gained per day from hatching until sampling (assuming the same mass at hatching for all individuals), which is a common procedure in ectotherms (e.g. [44,45]). To assess the effect of the temperature manipulation on growth rates we ran a linear mixed model (*lmer* function in package *lme4*) with *growth rate* as the dependent variable, *temperature* and *developmental stage* (plus their interaction) as independent variables, and *tank* nested within *clutch* as random factors. For telomere data, we explored the contribution of the factor *sex* on telomere length, but found it to be non-significant (overall effect: *χ* ^2^ = 1.29, 1 d.f., *p* = 0.255) so dropped it from further analyses.

In this study, we were mainly interested in investigating whether: (i) telomere length varies across tissues and developmental stages (particularly through metamorphosis), and in response to temperature conditions, (ii) the relationship between telomere length and growth rate depends on the tissue, the developmental stage and/or temperature conditions and (iii) the relationships between telomere lengths in different tissues within an individual vary across developmental stages. For (i), since not all tissues were present in all developmental stages, we conducted a linear mixed model for each tissue, with *relative telomere length* as the dependent variable, *developmental stage*, *temperature* and their interaction as independent factors, and *tank* nested within *clutch,* and *qPCR set*, as random factors. As plots suggested that liver and muscle telomeres may gradually shorten with age (similar to [46]), we additionally conducted two linear mixed models including liver or muscle *relative telomere length* as the dependent variable, *days from hatching* and *temperature* as independent variables, and *tank* nested within *clutch* as random factors. For (ii)*,* we conducted a linear mixed model for each developmental stage, with *relative telomere length* as the dependent variable, *organismal growth rate* (i.e. body mass gained per day from hatching until sampling)*, tissue type*, *temperature* and their interactions as independent variables, and *clutch, individual id* and *qPCR set* as random factors. In these analyses, we could not include liver and heart telomere lengths from individuals at stage NF54 because we pooled samples from two individuals, hence individual growth rates could not be calculated. We additionally used the same approach for analysing the relationship between body mass and relative telomere length. For (iii)*,* we calculated Pearson's correlations between telomere lengths of pairs of tissues at each sampling point, with the help of the function *ggpairs* (*GGally* package). Also, to explore the extent of individual variation in telomere lengths, we used the function *ranova* (package *lmerTest*) to obtain the significance of the random factor *individual id*, and the function *ICCest* (package *ICC*) to calculate intra-individual correlation coefficients between telomere lengths of an individual's tissues*.* We used the function *emmeans* (package *emmeans*) for *post hoc* Tukey tests, and the function *r.squaredGLMM* (package *MuMIn*) to calculate conditional and marginal *R* ^2^. Relative telomere length and organismal growth rate data were log-transformed to improve parametric assumptions.

## 3. Results

Survival did not vary between temperature treatments throughout the experiment (developmental stage by temperature interaction: *χ* ^2^ = 1.28, 4 d.f., *p* = 0.865; temperature effect: *χ* ^2^ = 0.01, 1 d.f., *p* = 0.983). The effect of temperature on growth rate was dependent on the developmental stage (developmental stage by temperature interaction: *χ* ^2^ = 12.59, 4 d.f., *p* = 0.014). A warmer temperature during larval development induced faster growth rates from hatching to stage NF54, NF60 and 70 day-old individuals (all *post hoc* test *p*-values < 0.004; electronic supplementary material, figure S2). By contrast, the temperature during the larval period had no significant impact on growth up to the NF66 or 7-month stage (*post hoc* test *p*-values = 0.066 and 0.190, respectively; electronic supplementary material, figure S2). Growth rate did not vary between sexes across developmental stages or in response to temperature (all *p* > 0.287).

**Figure 1.**
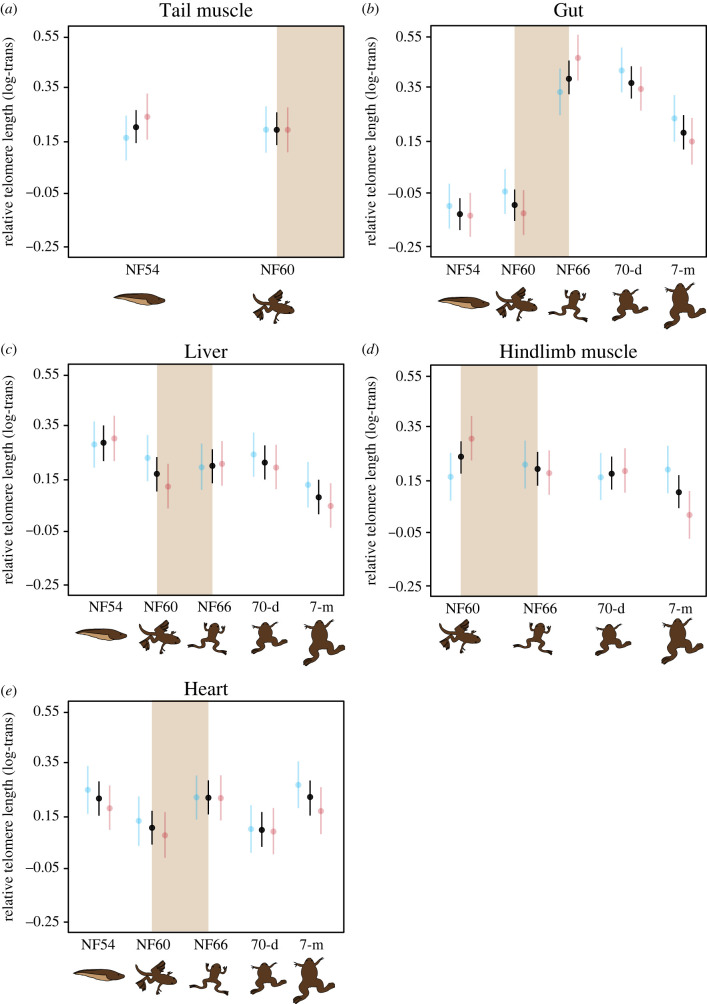
Relative telomere length (log-transformed) across tissues and developmental stages for *Xenopus laevis* from larval (tadpole) stage NF54 to 7-month-old frogs. Black dots and lines indicate overall mean and s.e. Blue and red dots and lines indicate estimated marginal means and s.e. in individuals exposed to a cold (19°C) or warm (23°C) temperature regime during the larval period, respectively. Temperature conditions were the same (19°C) for all individuals from NF60 onwards. The brown bands highlight the metamorphosis period. Drawings are non-scale representations of the appearance of individuals at each sampling point.

Telomere lengths across developmental stages showed different dynamics among tissues, whereas temperature had an overall negligible effect (table 1). In tail muscle, telomere lengths remained relatively constant throughout larval development (table 1; figure 1 *a*). By contrast, the average length of gut telomeres varied across developmental stages (table 1; figure 1 *b*), notably increasing during metamorphosis so that they were longer on average at the end of metamorphosis (stage NF66) than at pre-metamorphic stages (both Tukey-test *p*-values < 0.001). This difference was maintained after metamorphosis, such that while gut telomeres were not significantly shorter in adult frogs than they were immediately after metamorphosis (though the *p*-value was close to significance so perhaps a higher sample size would have given more power here, Tukey-test *p*-value = 0.059), they were still longer than in larval stages (all Tukey-test *p*-values < 0.002). Liver telomere lengths also varied across developmental stage (table 1; figure 1 *c*), mainly due to their being shorter in adults than in larvae (Tukey-test *p*-value = 0.020). A linear mixed regression including days from hatching suggests that liver telomeres gradually shortened with age (*χ* ^2^ = 7.43, d.f. = 1, *p* = 0.006). The length of hindlimb muscle telomeres did not differ significantly between samples taken at different developmental stages (table 1; figure 1 *d*) but was not significantly shorter with age (*χ* ^2^ = 3.48, d.f. = 1, *p* = 0.062). Finally, we did not detect any noticeable variation in heart telomere lengths across developmental stages (table 1; figure 1 *e*).

**Table 1.** Summary of the linear models testing for the effect of developmental stage and temperature on relative telomere lengths of five tissues in in *Xenopus laevis*. $R_{m}^{2}$ and $R_{c}^{2}$ refer to the variation explained by fixed, and fixed plus random terms, respectively. Bold indicates significant effects (*p* < 0.05).

	tail muscle			gut			liver^a^			heart			hindlimb muscle^a^		
	$R_{m}^{2}$ = 0.01, $R_{c}^{2}$ = 0.67			$R_{m}^{2}$ = 0.27, $R_{c}^{2}$ = 0.56			$R_{m}^{2}$ = 0.03, $R_{c}^{2}$ = 0.37			$R_{m}^{2}$ = 0.03, $R_{c}^{2}$ = 0.50			$R_{m}^{2}$ = 0.03, $R_{c}^{2}$ = 0.42		
	d.f.	*χ* ^2^	*p*-val	d.f.	*χ* ^2^	*p*-val	d.f.	*χ* ^2^	*p*-val	d.f.	*χ* ^2^	*p*-val	d.f.	*χ* ^2^	*p*-val
developmental stage	1	0.01	0.906	4	103.38	**< 0.001**	4	10.73	*0.030*	4	7.07	0.132	3	3.52	0.318
temperature	1	0.17	0.681	1	0.20	0.652	1	0.79	0.373	1	0.26	0.611	1	0.01	0.947
developmental stage x temperature	1	0.51	0.476	4	2.90	0.573	4	0.76	0.943	4	1.79	0.940	3	4.46	0.216

^a^See §3 for further analyses.

**Figure 2.**
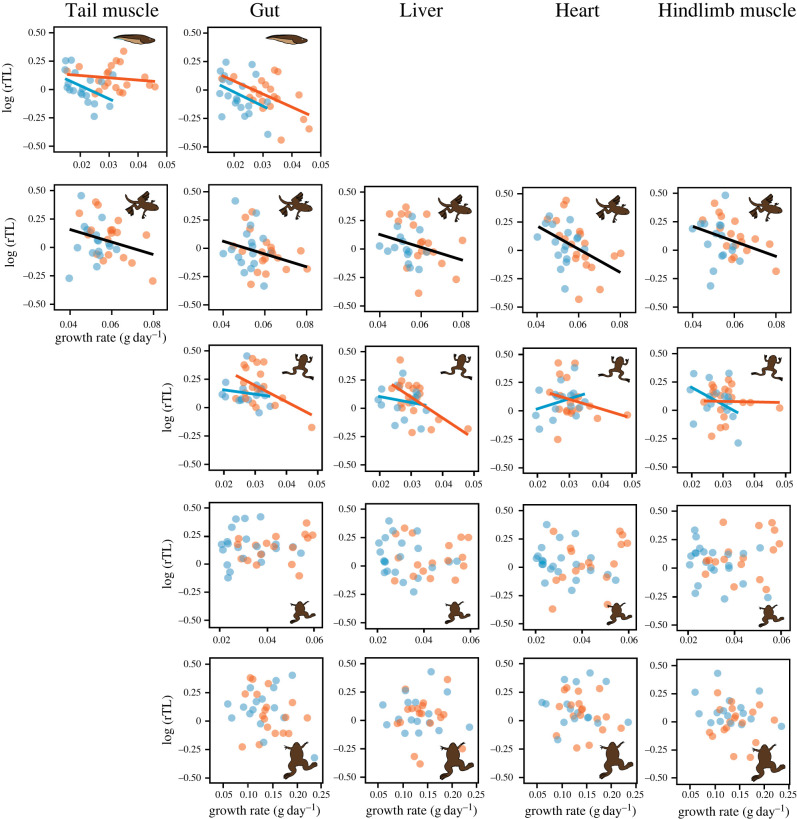
Linear correlations between relative telomere length (log-transformed) and an individual's whole-body growth rate across tissues and stages of development in *Xenopus laevis*. Growth rate was calculated as the mean gain in mass/day from hatching until the time of sampling. Blue and red dots and lines indicate values and the slope of the regressions in individuals exposed during the larval period to a cool (19°C) or warm (23°C) temperature regime, respectively. Regression lines are shown when an overall (growth rate or temperature) or interaction effect was significant (table 2). Temperature conditions were the same (19°C) for all individuals from NF60 onwards. Drawings are non-scale representations of the appearance of individuals at each sampling point.

The relationship between an individual's telomere length and its growth rate varied from larval to post-metamorphic stages. In the early larvae (stage NF54), this relationship was tissue- and temperature-dependent: telomeres were shorter in individuals that had been growing faster under the cool temperature regime, a pattern that was more marked in the tail (table 2; figure 2 *a*). By the time of the onset of metamorphosis (stage NF60), telomere length showed, overall, a strong negative relationship with growth rate across all sampled tissues and in both temperature regimes (table 2; figure 2 *b*). By the end of metamorphosis (stage NF66), the relationship between telomere length and growth rate was again tissue- and temperature-dependent (table 2; figure 2 *c*), but in later life all relationships between telomere length and growth rate became non-significant (table 2; figure 2 *d,e*). The relationship between body mass and relative length was similar than for growth rate (electronic supplementary material, table S2).

**Table 2.** Summary of linear models testing for the effect of growth rate, temperature, and tissue on relative telomere length in *Xenopus laevis* from stage NF54 larvae to 7-month-old adults*.* Metamorphosis took place between stages NF60 and NF66. $R_{m}^{2}$ and $R_{c}^{2}$ refer to the variation explained by fixed, and fixed plus random terms, respectively. Bold indicates significant effects (*p* < 0.05).

	54-NF stage			60-NF stage			66-NF stage			70-day			7-month		
	$R_{m}^{2}$ = 0.23, $R_{c}^{2}$ = 0.73			$R_{m}^{2}$ = 0.18, $R_{c}^{2}$ = 0.69			$R_{m}^{2}$ = 0.10, $R_{c}^{2}$ = 0.65			$R_{m}^{2}$ = 0.08, $R_{c}^{2}$ = 0.68			$R_{m}^{2}$ = 0.05, $R_{c}^{2}$ = 0.68		
	d.f.	*χ* ^2^	*p*-val	d.f.	*χ* ^2^	*p*-val	d.f.	*χ* ^2^	*p*-val	d.f.	*χ* ^2^	*p*-val	d.f.	*χ* ^2^	*p*-val
growth rate	1	8.61	**0.003**	1	18.82	**<0.001**	1	1.22	0.268	1	0.05	0.827	1	0.10	0.760
tissue	1	32.76	**<0.001**	4	41.74	**<0.001**	3	17.44	**<0.001**	3	29.66	**<0.001**	3	7.48	0.058
temperature	1	6.24	**0.012**	1	1.96	0.162	1	0.27	0.602	1	0.26	0.609	1	1.25	0.263
growth rate x tissue	1	4.93	**0.026**	4	3.57	0.468	3	1.13	0.770	3	1.08	0.783	3	1.19	0.756
growth rate x temperature	1	0.49	0.482	1	0.07	0.794	1	0.71	0.398	1	0.01	0.934	1	1.05	0.307
tissue x temperature	1	0.01	0.982	4	6.21	0.184	3	1.18	0.757	3	1.01	0.799	3	0.93	0.818
growth rate x tissue x temperature	1	0.77	0.381	4	1.23	0.873	3	8.73	*0.033*	3	1.81	0.612	3	1.77­­	0.621

Pearson coefficients for the relationship between telomere lengths in pairs of tissues from the same individual were always positive and relatively high across development stages (electronic supplementary material, table S3). The random factor ‘individual’ was significant in the model examining the relationship between tissue telomere lengths and growth rate across developmental stages (all *p*-values of likelihood *ranova* tests < 0.049, except in NF54 larvae where *p*-value = 0.084). Intraclass correlation coefficients examining the relationships among telomere lengths of an individual's tissues at each developmental stage were 0.41, 0.46, 0.35, 0.38 and 0.47 in NF54, NF60, NF66, 70-day and 7-month individuals, respectively.

## 4. Discussion

Despite a growing interest in telomere dynamics in ectotherms [47], this is, as far as we are aware, the first study of telomere dynamics across the period of metamorphosis, a life transition undergone by the majority of animals. Metamorphosis requires complex transformations, including rapid cell differentiation and tissue growth, all processes thought to affect telomere dynamics [15,48]. As predicted, telomere dynamics were linked to the degree of transformation experienced by tissues from larva to adult stage, and organismal growth shaped telomere lengths until metamorphosis. Gut telomeres experienced the largest change in average length, mostly during metamorphosis, as predicted given the high degree of gut tissue remodelling. By contrast, liver and hindlimb muscle telomeres mostly shortened at the adult stage compared to larvae, whereas heart and tail muscle telomeres remained unaltered. The relationship between organismal growth rate and telomere length was negative across tissues until metamorphosis, but these negative relationships completely disappeared from that point onwards. Temperature during the larval period affected growth and developmental rates, but had only a negligible impact on telomere dynamics, and had no carry-over effects in post-metamorphic telomeres. Finally, an individual's telomere lengths showed strong correlations across tissues for all the studied developmental stages. Overall, our study indicates that telomere lengths and their relationship with organismal growth are governed in *X. laevis* by the tissue-specific cellular processes experienced during the change from larva to adult frog.

In our study, the telomeres of gut cells showed a remarkable change in length during metamorphosis, presumably linked to the degree of remodelling experienced by this tissue. During *X. laevis* metamorphosis, gut shortening and remodelling includes larval epithelial apoptosis and differentiation into stem cells [27,28,49], a phase of cell turnover that may explain longer telomeres in the gut cells of metamorphic individuals than in larvae. After metamorphosis, a self-renewing system of stems cells is established in the intestinal epithelium and differentiated cells invaginate into connective tissue to form the intestine folds [27,28,49]. This system is present in adult but a large degree of differentiation may be behind the telomere shortening in gut tissue that occurs from the conclusion of metamorphosis through to the adult frog. By contrast, tissues undergoing a much lower degree of transformation during metamorphosis, such as the liver and hindlimb muscle, experienced telomere shortening from the larval to the adult stage. The frog liver experiences its main structural changes in early larval life and only undergoes minor functional transformations during metamorphosis [11]. However, it greatly increases in size from larva to adult, and thus cell division may have driven telomere shortening in this tissue. In hindlimbs, muscle development is characterized by a myogenesis that leads to the formation of adult multinucleated myofibres through myoblast fusion and may include the differentiation of some stem cells [29,31,32], which may explain why telomeres only shortened slightly in this tissue. It is noteworthy that, despite hindlimb muscle first appearing at a relatively late larval stage, its telomeres had a similar length to that of all the other tissues (which had been formed earlier), likely because the muscle is mostly formed from existing myoblasts. The exception are gut telomeres, being longer than those in the other tissues across all post-metamorphic developmental stages (except in adults, where extensive differentiation has already taken place), likely linked to the presence of a self-renewal system of gut stem cells. Low cell-division rates but also the regenerative capacity of the heart in *X. laevis* (at least until frogs are six months old [30]) and the underlying mechanisms of that process might explain the lack of variation in heart telomere lengths over time. The expression of telomerase has been described as essential for heart regeneration in zebrafish [50] and is known to be active in the heart of adult *X. laevis* [51], thus this enzyme may have compensated for telomere loss caused by cell division. Finally, the fact that the tail muscle only experiences small changes in size between the two developmental stages at which we measured tail telomere lengths may underlie the absence of a change in telomere lengths recorded in this tissue.

Processes such as *de novo* formation of stem cells and the subsequent increase in average telomere length in the metamorphic gut support the idea that metamorphosis can act as a rejuvenating process in some tissues. Indeed, the gut is a central organ in ageing and the length of its telomeres has been shown to have systemic effects, including on lifespan [52]. The tissue-specific expression of mechanisms that buffer damage on telomeres (e.g. ROS scavengers) or maintain their sequence (telomerase; [51]) could have also played a role in explaining the small amount of telomere shortening observed in post-metamorphic individuals. Intriguingly, the correlation in telomere lengths among tissues of the same individual was, overall, high throughout life stages. While several studies have found positive and strong correlations between telomere lengths in different tissues of the same animal (e.g. [53–57]), our study shows that those correlations do not necessarily indicate similar telomere dynamics. Further research should explore whether the rate of telomere change is similar in tissues of adult frogs across age, as for example found in fully developed humans [58].

Metamorphosis is considered to be a vulnerable life-history stage since it is metabolically expensive and also exposes individuals to new environmental conditions that can influence their fitness [6]. These characteristics likely explain why larger individuals at metamorphosis normally show higher fitness [20,59]. Telomere loss is often suggested as an indicator of the costs of growth, based on the premise that both oxidative stress and the number and the rate of cell division accelerate telomere attrition [15,16,60,61]. In our study, the relationship between telomere length and growth rate was generally negative until metamorphosis, whereas a lack of correlation was observed at the juvenile and adult stages. Given the importance of reaching a large size at metamorphosis, the existence of shorter telomeres in faster growing larvae may be understood as an adaptive resource allocation from telomere maintenance to organismal growth, similar to what is found in juvenile Atlantic salmon with shorter telomeres, which have a higher survival to spawning [62]. However, the existence of maintenance costs of telomeres is so far a hypothesis supported by correlational data. Also, having shorter telomeres early in life may have detrimental implications in the long term, which needs to be explored in amphibians. Temperature conditions also influenced the relationship between telomere length and growth until metamorphosis. Larvae had longer telomeres for a given growth rate if living in warm conditions than if in the cool treatment. This suggests that larval growth was more costly in the colder environment. A lower temperature could cause this effect for the following two reasons. (i) It will decrease the maximum rate of food uptake so will create greater resource limitations, i.e. growth can only be achieved through reduced investment in cellular maintenance/repair, including of telomeres [63,64]. (ii) Lower temperatures also often cause a decrease in mitochondrial respiration rate [65], so that individuals may be able to maintain ATP production only at the expense of an increased production of ROS [66,67], which can shorten telomeres. This pattern is analogous to the telomere dynamics observed in juvenile Atlantic salmon *Salmo salar*, where those in a harsher environment had shorter telomeres for a given growth rate than those growing in more benign conditions [68]. The available evidence shows equivocal effects of temperature on ectothermic telomeres (e.g. [53,68–70]). Species such as *X. laevis*, which inhabit environments where temperatures can fluctuate over short time periods, may have evolved buffering mechanisms to compensate for the costs of altering mitochondrial respiration rates [71]. Further research is needed to understand the potential role of global warming in shaping the relationships between developmental, growth rate and ageing rates in ectothermic species [24], information that may be incorporated into conservation actions and species distribution models at different scales [72].

Our study shows for the first time that metamorphosis is associated with strikingly contrasting changes in telomere lengths in different tissues. Overall, these results highlight the complexity of telomere dynamics in organisms undergoing metamorphosis, which may even act as a rejuvenating mechanism, in contrast with the age-related pattern of telomere shortening often found in tissues of non-metamorphosing individuals. We hope this study will encourage future research exploring the relationship between tissue-specific telomere dynamics, ageing rate, fitness and the mechanisms involved in telomere maintenance in species with complex life cycles.

## Data Availability

Data supporting this article are available at https://doi.org/10.6084/m9.figshare.21688343 [73]. The data are provided in the electronic supplementary material [74].
